# One-pot synthesis of graphene- cobalt hydroxide composite nanosheets (Co/G NSs) for electrocatalytic water oxidation

**DOI:** 10.1038/s41598-018-32177-9

**Published:** 2018-09-13

**Authors:** Robab Mehmood, Neelam Tariq, Muhammad Zaheer, Fozia Bibi, Zafar Iqbal

**Affiliations:** 1 Department of Chemistry and Chemical Engineering, SBA School of Science and Engineering, Lahore University of Management Sciences (LUMS), Lahore, 54792 Pakistan; 2 Department of Chemistry, University of Poonch Rawalakot (UPR) Rawalakot, 12350 Azad Jammu and Kashmir, Pakistan

## Abstract

We report a one-pot method for the preparation of graphene-cobalt hydroxide nanosheets (Co/G NSs) and their use as an effective elelctrocatalyst for water oxidation. Mechanical exfoliation of graphite via sonication produced graphene sheets, which were stabilized by the surface adsorption of a cationic surfactant (CTAB). In a subsequent step, varying amount of a cobalt complex [sodium hexanitrocobaltate(III)] was added which selectively bound with the positively charged head of surfactant. In the last step, cobalt complex was reduced with sodium borohydride to obtain Co/G NSs catalyst. The catalyst showed lower overpotential (280 mV) as compared to benchmark catalysts and decent stability and turnover frequency (TOF: 0.089 s^−1^) for oxygen evolution reaction (OER).

## Introduction

The present energy resources are insufficient to fulfill global energy in near future due to their rapid depletion, therefore new technological advances to quest clean, sustainable and viable energy resource are highly demanded^1–3^. One of the renewable and alternative energy sources is water, which could undergo photochemical and electrochemical splitting to produce oxygen and hydrogen green fuel^4,5^. In comparison to two-electron hydrogen evolution reaction (HER)^6,7^, four-electron oxygen evolution reaction (OER) possesses sluggish kinetics requiring higher overpotential (*η*) for the achievement of benchmark 10 mA/cm^2^ current density^8^. In the past decade various molecular^9^ and heterogeneous catalysts^10–12^ have been developed to achieve oxidation of water at lower overpotential. Focus has been on the earth-abundant metal catalysts^13–15^ that could possibly replace the benchmark OER catalysts based on scarcely available Pt, Ir and Ru^16^. Among other abundantly available transition metals, much attention has been gained by Co catalyst since the pioneer work of Nocera^17^ and several catalysts based on oxides, chalcogenides, phosphides and phosphates of cobalt have been developed for water splitting^18–22^ and energy storage^23–27^. However these catalysts based on earth-abundant metals need to be improved to achieve low overpotentials, low Tafel slopes, high Faraday efficiency, high turnover frequency (TOF) and stability^28^. Among others, carbon^29^ and graphene-supported cobalt heterostructures have shown high current density, low overpotential and significant stability for OER^30–35^. Graphene, a hexagonally arranged two dimensional material made up of sp^2^-carbon atoms possesses extraordinary properties like room temperature electron mobility, high thermal conductivity and ability to withstand high current densities^36^. Very high mechanical strength, surface area (>2600 m^2^/g) and density of surface active sites make graphene particularly suitable for catalysis^37–39^. Electrochemical properties of graphene greatly depend upon the synthetic method and can potentially be enhanced by the incorporation of heteroatoms in the graphene lattice which modulates the spin density and charge distribution of carbon atoms thereby creating the catalytically active sites for OER^32,40–42^. However conventional synthesis of graphene by Hummer’s method^43^ and its modifications^44,45^ involves the use of hazardous chemicals or their products whose removal, reuse and disposal is an issue. Chemical vapor deposition (CVD)^46–50^ and liquid-phase exfoliation (LE)^51–53^ are the only alternative (green) methods^54,55^ for the fabrication of highest-quality graphene known for its superior properties. The former method however, is still relatively expensive as it demands large energy and the removal of the substrate^36^. Recently materials based on cobalt(II) hydroxide has shown potential in electrochemical water splitting^56–58^. However poor electrical conductivity of the material needs to be improved in order to obtain a significant OER activity^59–63^. Inspired by the pioneer work of Coleman *et al*.^64–68^ we report a one-pot procedure for the solution-phase synthesis^69^ of graphene-Co(OH)_2_ nanosheets (Co/G NSs) which catalyze water oxidation at remarkably low overpotential of 280 mV providing high TOF of 0.089 s^−1^.

## Results and Discussion

**Figure 1:**
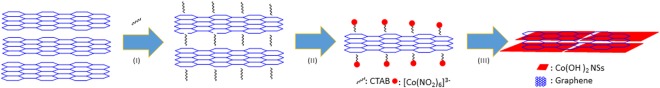
Schematic presentation for the synthesis of Co/G NSs. (I) Mechanical exfoliation of graphite in the presence of CTAB; (II) Cobalt complex electrostatically binds with N-functions of the surfactant; (III) Reduction of Co ion to Co/G NSs.

We used cationic surfactant, cetyltrimethyl ammonium bromide (CTAB), to produce graphene flakes in water by mechanical exfoliation of graphite via ultrasonication (Fig. 1)^65^. The surfactant gets adsorbed on the surface of so produced graphene via Van der Wall’s interactions and stabilizes the 2-dimensional material (I in Fig. 1). In the following step, a cobalt complex (Na_3_[Co(NO_2_)_6_]: CoL) was added whose anion ([Co(NO_2_)_6_]^3-^) electrostatically binds with the the positive head of the surfactant (II in Fig. 1). In third step, cobalt complex was reduced to produce Co/G NSs (III in Fig. 1). In past, stable graphene dispersions with an optimum yield have been achieved with a maximum surfactant concentration of 0.1 mg/ml^70^. Any higher concentration of surfactant leads to the agglomerations of graphene flakes due to the disruption of electric double layer by counter ions^71^.

**Figure 2:**
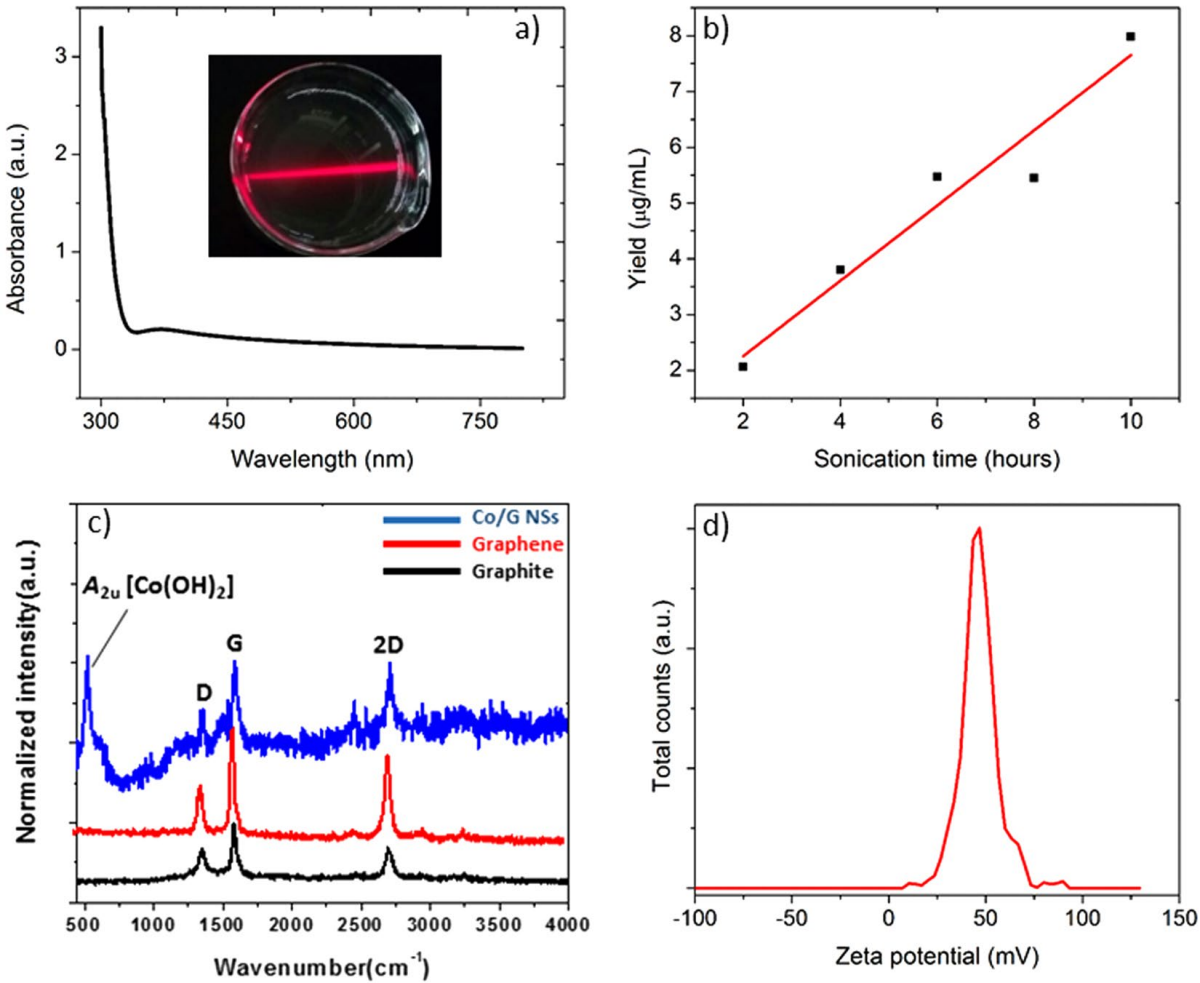
(**a**) Absorption spectrum of CTAB-supported graphene. Tyndall effect shown by graphene dispersions is shown in inset. Surfactant absorption is dominated below 400 nm while any absorption above attributes to suspended graphene. (**b**) Graphene yield as the function of sonication time; CTAB concentration (C_CTAB_): 0.1 mg/mL; initial graphite concentration (C_G_) of 3 mg/mL. (**c**) Raman spectra (graphite, graphene and Co/G NSs) and zeta potential (**d**) of graphene synthesized by liquid-phase exfoliation of graphite.

UV-vis absorption spectrum of graphene dispersion was found featureless and flat (Fig. 2a) at and above 400 nm where absorption by surfactant molecules was negligible. Absorption at 660 nm could therefore be attributed to suspended graphene and was used to find the concentration using Beer-Lambert law (A = αC*l*)^71^. For the calculation of extinction coefficient (α), a graphene dispersion (500 ml) was prepared using graphite (3 mg/mL) and CTAB (0.1 mg/mL). After centrifugation and decantation, absorption spectrum was taken at 660 nm. 60 mL of this dispersion was filtered through pre-weighted filter units, dried and weighed to find the graphene concentration. The value of absorptivity constant was found to be 1256 mL/mg/*l* which is close to the reported value of 1390 mL/mg/*l*^65^.

Next, various parameters for instance, sonication time, surfactant and graphite concentration were optimized to improve graphene yield. It was found that graphene yield increases almost linearly with sonication time as presented in Fig. 2b. The yield (8 μg/mL) obtained after ten hours of sonication was used as the optimum value for the rest of the experiments. In the same way concentration of graphite (C_G_) and CTAB (C_CTAB_) were also optimized and optimum yield of graphene was found with C_G_ = 3 mg/mL and C_CTAB_ = 0.1 mg/mL [see Fig. S1 in supporting information (SI)]. Under optimized conditions, 500 mL of graphene dispersion was centrifuged (@15000 rpm for 1 hour) followed by vacuum filtration to remove undissolved graphite. Afterwards graphene concentration in the dispersion was calculated using uv-visible spectrophotometry^65^.

Raman spectroscopy^66,72^ was used to get further structural information about graphite, graphene and Co/G NSs materials. All the spectra as presented in Fig. 2c contain D (~1352 cm^−1^), G (~1587 cm^−1^) and 2D (~2710 cm^−1^) bands^65^. The D band corresponds to the breathing modes of sp^2^ atoms in rings^73^ while G band attributes to in-plane vibrational modes of sp^2^-bonded carbon atoms^74^. 2D band which is the second order of D band appeared at ~2710 cm^−1^ in all the spectra. Intensity ratio of G and 2D bands (I_G_/I_2D_) can be used to differentiate graphite from graphene and is probably the best way to decide layer-thickness^75^. I_G_/I_2D_ ratio in our case decreased from 1.42 in graphite to 1.2 in graphene and to 1.0 in Co/G NSs material. The latter corresponds roughly to a thickness of 5-layers^76^. Raman spectrum of Co/G NSs showed an additional intense band at ~520 cm^−1^ which was assigned to *A*_2u_ lattice mode of cobalt(II) hydroxide^77–79^.

Stability of graphene dispersions was tested by zeta potential analysis (Fig. 2d) as these colloids were expected to be stabilized by electrostatic repulsion between the surfactant-coated graphene sheets. Hydrophobic tail of CTAB gets adsorbed on graphene sheets due to Van der Waal forces and impart positive charge due to the positively charged head of surfactant^80^. The formation of so-called electric double layer will avoid possible agglomeration of graphene sheets^81^. It is reported that colloidal particles will be electrostatically stabilized if the zeta potential values fall between −15 mV and 15 mV^82^. Our graphene dispersion showed a zeta potential value of 47 mV which indicated stability of the system^83^.

**Figure 3:**
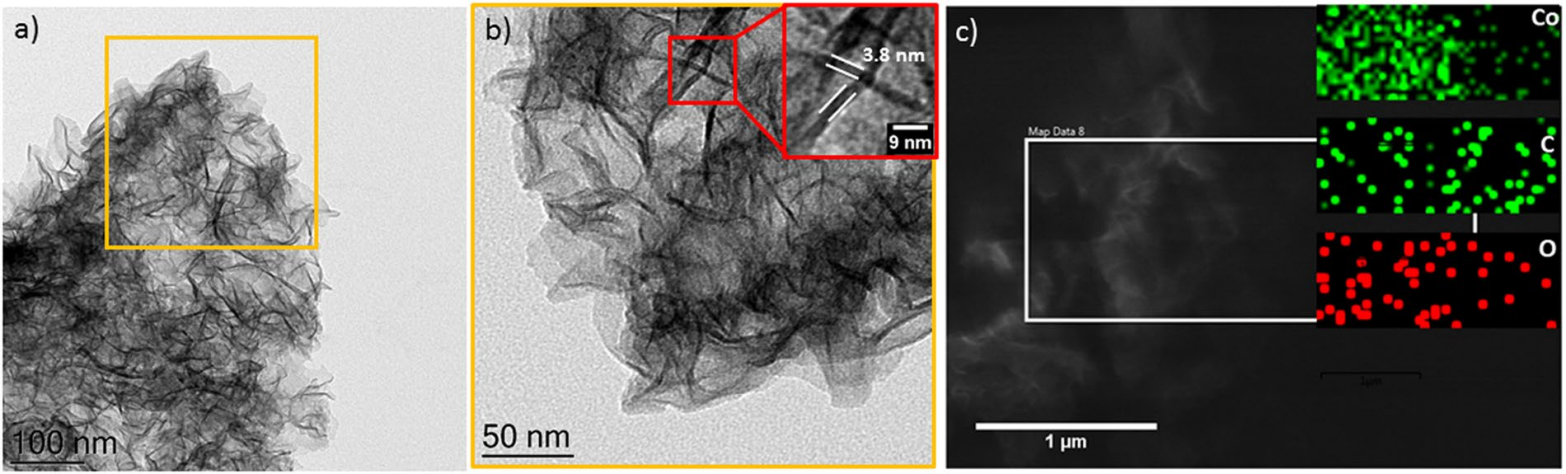
Transmission Electron Microcopy (TEM) images of Co/G NSs catalyst. Inset in (**b**) shows a magnified view of a selected portion. SEM with EDX mapping of the selected region is presented in (**c**).

The morphology of Co/G NSs was analysed by TEM. Figure 3a presents an overview image of the Co/G NSs material showing a sheet-like morphology. At a higher resolution (Fig. 3b) folded nanosheets (NSs) with a thickness of ~3.8 nm (inset) were observed. A SEM image of the material is presented in Fig. 3c where again folded NSs can be clearly seen. EDX maps of the selected region suggest the presence of Co, O and C with an atomic ratio of 52, 25 and 9 atomic percent. Therefore we conclude that NSs are made up of either oxides or hydroxides of Co. EDX spectrum of another region is presented in S2 (SI) provides further confirmation about the chemical nature of NSs.

**Figure 4:**
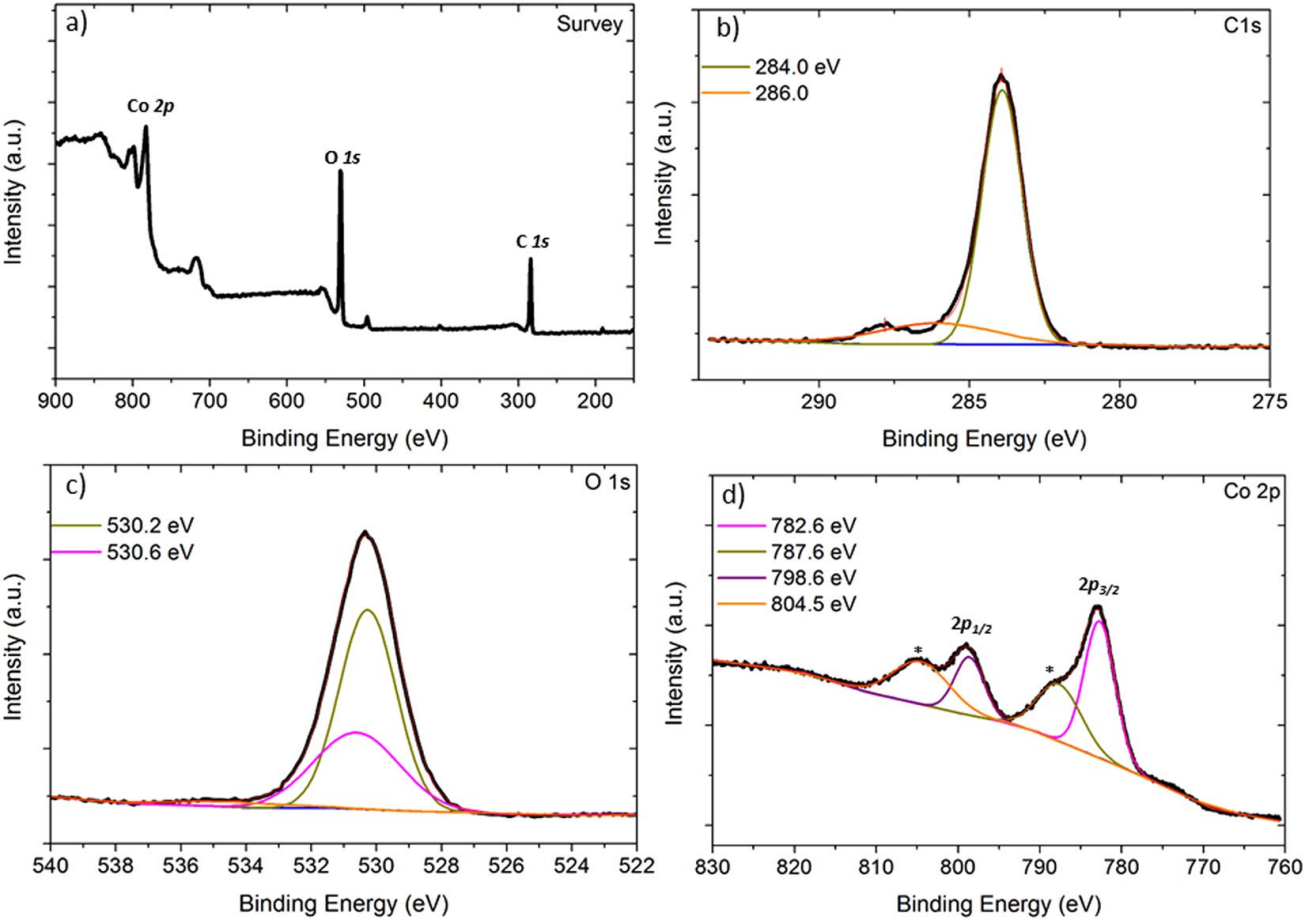
XPS spectra of Co/G NSs material. (**a**) Survey spectrum and high resolution spectra for O1s (**b**), C1s (**c**) and Co2p (**d**).

Co/G NSs material was further confirmed by X-ray photoelectron spectroscopy (XPS). In survey spectrum of the material presented in Fig. 4a, peaks at 284.0, 531.0 and 779 eV were indexed to C(1s), O(1s) and Co(2p) respectively. Magnified view of C(1s) spectrum is provided in Fig. 4b where a main peak at 284.0 was assigned to *sp*^2^ carbon and a small peak at higher binding energy (286.0 eV) to O-C-O and C-OH groups^84^. The oxygen (1 s) core spectrum (Fig. 4c) was deconvoluted into two peaks at 530.2 and 530.6 eV and were assigned to O(1 s) in cobalt hydroxide Ca(OH)_2_^85^.

High resolution XPS spectrum of Co 2p (Fig. 4d) showed a spin-orbit splitting into 2p_3__/2_ and 2p_1/2_ with an energy difference of ~16 eV typically known for cobalt(II) hydroxide^86,87^. 2p_3/2_ peak was deconvoluted into two peaks centering at 782.6 and 787.6 eV and were assigned to Co(OH)_2_, the latter being the shake-up satellite of 2p_3/2_^88^. The peak at higher binding energy was broken down into two Gaussian peaks at 798.6 and 804.5 eV and were attributed to 2p_1/2_ and its shake-up satellite for cobalt hydroxide respectively. Although Co 2p_3/2_ peak of Co_3_O_4_ also occurs in the same region (~779 eV), its presence in the sample was ruled out because of the fact that Co_3_O_4_ does not show any satellite peak^89^.

**Figure 5:**
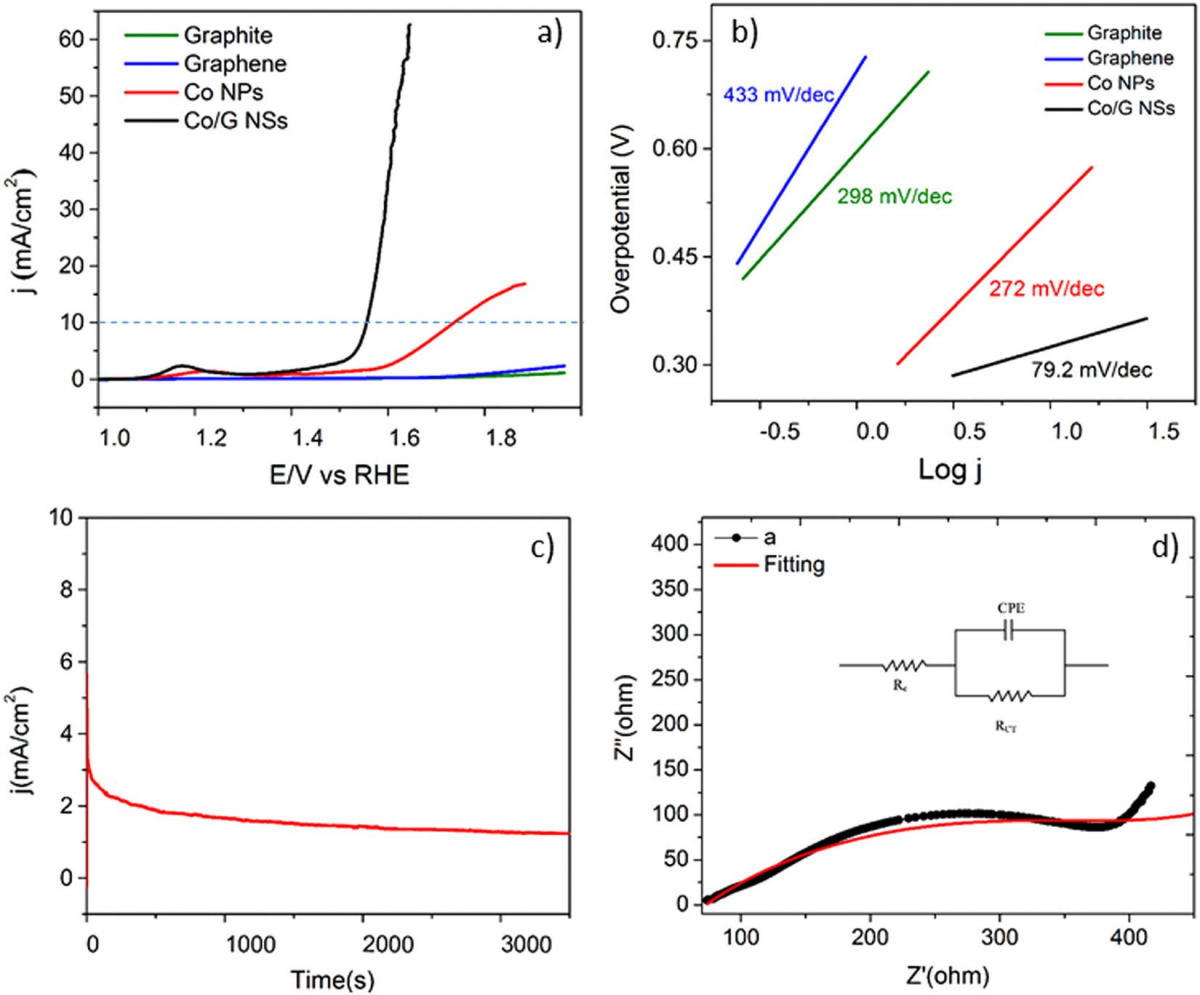
LSV polarization curve (**a**), Tafel plot (**b**), chronoamerpmetric plot (**c**) and Nyquist plot (**d**) of Co/G NSs catalyst. Circuit diagram is shown in (**d**) that was applied over the EIS data.

Co/G NSs composite for oxygen evolution reaction (OER) was tested in 0.1 M KOH using a three-electrode system details of which are given in experimental part. Linear sweep voltammetry (LSV) of the material revealed an efficient OER catalytic activity (onset 1.51 V) with a low over potential of 280 mV to achieve 10 mA/cm^2^ current density (Fig. 5a). This OER activity (280 mV) of Co/G NSs is even better than the benchmark IrO_2_ catalyst (297 mV) under the same experimental conditions^90^ and other cobalt-based catalysts reported to date (see Table S1).

For electrocatalytic performance, Tafel slope can be used for the kinetics study to determine the rate and rate determining step during OER reaction. We calculated a value of 79.2 mV/dec for Co/G NSs material (see Fig. 5b) which is though high as compared to other first-row transition metals, nevertheless provides valuable information about the kinetic parameters. Value of Tafel slope suggests that the formation of MO (where M denotes a free metal site) from the adsorbed OH^−^ ions is the rate determining step^91^. The mass activity (583.3Ag^−1^) and TOF (0.089 s^−1^) of Co/G NSs catalyst were also found to be high (Table S2) at low mass loading (17 µg/cm^2^).

Stability of the catalysts was evaluated by controlled current electrolysis (CCE) experiment performed in 0.1 M KOH (pH = 13). Chronoamperometry was conducted at 1.65 V (vs RHE) while maintaining current density of 5.65 mA/cm^2^ for 3600 sec under steady state condition. From the stability plot (Fig. 5c), the tolerance of this electrocatalyst against intermediate species may be attributed the strong interaction of Co with graphene. Fig. S3 displays a plot under constant current density of 10 mA/cm^2^ between applied potential (E/V vs RHE) and time (t) and it shows that this catalyst is stable up to 1100 sec vs applied current density.

Electrochemical impedance spectroscopy (EIS) measurement was also carried out to investigate OER activity of Co/G NSs. Figure 5d shows the Nyquist plot at applied potential of 1.51 V (vs RHE) in the frequency range of 1 Hz to 100 kHz to determine the conductivity and resistivity of the catalysts. The semicircular portion of the plot provided solution resistance (Rs), charge transfer resistance (R_ct_), and double layer capacitance (C_dl_). R_ct_ value (408.7Ω) was rather high for our catalyst system and could be attributed to the presence of surfactant. Solution resistance (R_s_) was calculated to be 72.8Ω (Table S3).

Using C_dl_ value we calculated electrochemically active surface area of Co/G NSs to be 27 cm^2^ which can be correlated to the presence of more active sites at electrolyte/analyte interface, and hence higher electrocatalytic activity^92^ of our electrocatalyst as compared to other electrocatalysts (Table S2). There can be error in the reported value of specific capacitance of metal electrodes as large as 7% in acidic and basic media^11^.

The above results revealed an accessible and simple way to obtain stable dispersions of graphene in surfactant (CTAB)/water systems followed by the generation of Co(OH)_2_ NSs to fabricate a Co/G nanocomposite. The dispersion and concentration of graphene increased linearly with CTAB concentration and sonication time. Presence of graphene increased the electrical conductivity of Co(OH)_2_ and hence OER activity of the composite material. Current density of 63 mA/cm^2^ was achieved at low overpotential (280 mV) in basic medium. Electrocatalytic activity of Co/G NSs was found better as compared to the state-of-the-art catalysts based on the oxides of Ir and Ru. Overpotential value, ECSA, mass activity and TOF of the catalyst was also found superior than other cobalt-based catalysts reported so far.

## Methods

Graphite obtained from used zinc-carbon batteries was soaked and washed with hydrochloric acid to leach metal impurities. Cetyl trimethylammonium bromide (CTAB), Sodium borohydride (NaBH_4_), Sodium hexanitrocobaltate(III) (Na_3_[Co(NO_2_)_6_] was purchased from Sigma Aldrich. All the solutions were prepared in deionized water (DI) and methanol.

Uv-visible spectra were obtained on UV-1800 SHIMADZU Spectrophotometer while zeta potential was measured on Malvern ZETA SIZER NANO. Energy dispersive X-ray spectroscopy (EDX) and surface morphology were examined by using FEI NOVA nano SEM 450 equipped with Oxford EDX detector. Raman spectra were taken using Raman Microscope Renishaw, UK. Surface analysis of the catalysts was conducted using X-ray photoelectron spectrometer (ESCALAB 250Xi, Thermo Scientific, UK). Charging of the spectra was corrected against the graphitic-like C 1 s peak (from the instrument itself) taken at 284 eV and used as a reference. CASAXPS software (version 2.3.16) was used for peak fitting.

Transmission electron microscopy (TEM) was carried out by using a Varian LEO 9220 (200 kV) and a JOEL JEM-2200FS instrument. The sample was suspended in chloroform and sonicated for 5 min. Subsequently a drop of the suspended sample was placed on a grid (Plano S 166-3) and allowed to dry.

Electrochemical analysis was executed by using Gamray Reference 600 Potentiostat/galavanostat/ZRA with three-electrode system.

### Catalyst synthesis

Graphene was prepared by a simple mechanical exfoliation method. Typically, graphite and CTAB were mixed in 3.3:1 mass ratio in DI water and the mixture was sonicated for 10 hr in ultrasonic bath (Elma X-tra 70 H) whose temperature was kept below 50 °C. CTAB-stabilized graphene suspension was centrifuged (Centrifuge -5810R) for one hour to remove undissolved graphite and unstable graphene. In the next step, Cobalt complex was added to graphene dispersion (Co to graphene mass ratio = 1:100) and sonicated for 30 min. Afterwards, freshly prepared solution of NaBH_4_ was added and dispersion was stirred at room temperature for 2 hours.

For characterization, graphene dispersion was filtered through syringe filter (0.22 μm) and vacuum dried. For TEM and Raman analysis, dispersions were precipitated at 14000 rpm for 30 minutes.

### Electrochemical measurements

All the electrochemical experiments were performed at room temperature (25 °C) using standard three-electrode system. A glassy carbon (0.07 cm^2^) electrode (GCE) was scrubbed with alumina powder (0.05 µm) before using as the working electrode. A silver/ silver chloride electrode (Ag/AgCl) was used as reference electrode and platinum wire as counter electrode. All the potentials were converted into RHE by using following Nernst equation.

$${E}_{({\rm{RHE}})}={E}_{(\mathrm{Ag}/\mathrm{AgCl})}+(0.059\,\ast \,{\rm{pH}})+0.197{\rm{V}}$$

5 µL of prepared catalyst without any binder, was deposited on GCE and air dried at room temperature. Then all OER tests were achieved in the 0.1 M KOH at the scan rate of 20 mV/sec with a scan limit of 0 to 1 V. For the evaluation of electrocatalytic activity, all the calculation were made from the *i*R corrected voltammogram (Fig. 5)

Tafel slope was calculated by overpotential (η) and current density (j) by using Tafel equation.

$${\rm{\eta }}={\rm{b}}\,\mathrm{log}\,{\rm{j}}+{\rm{a}}$$

where ‘b’ is the Tafel slope and ‘a’ is the constant.

Theoretically, mass activity was calculated by using the following equation.

$${\rm{Mass}}\,{\rm{activity}}({{\rm{Ag}}}^{-1})={\rm{j}}/{\rm{m}}$$

where ‘j’ is the current density (mA/cm^2^) and ‘m’ is the loading mass of electrocatalyst (0.017 mg/cm^2^).

The turnover frequency (TOF) can also be theoretically calculated by considering that every metal atom is involved in catalysis.

$${\rm{TOF}}\,({\sec }^{-1})={\rm{j}}\times {\rm{S}}/4{\rm{nF}}$$

where S is the electrode surface area, F is the Faraday constant (96485.3 Cmol^−1^) and n is the number moles of electrocatalyst.

Electrochemically surface area (ECSA) for each system can be measured from double layer charging capacitance of electrocatalytic surface determined from non-faradic region of cyclic voltammetry at multiple scan rates from 10 mV/sec to 200 mV/sec of cyclic voltamogram^93–96^.

It is assumed that non- faradic current is due to double layer charging capacitance (Figs S4 and S5). A plot of scan rate and current was used for calculating double layer charging capacitance (C_dl_) which yield a straight line equation with a slope which is equal to C_dl_ according to following equation^97^ (Fig. S6).

$${{\rm{i}}}_{{\rm{c}}}={v{\rm{C}}}_{{\rm{dl}}}$$

where i_c_ is the double layer charging current, *v* is the scan rate and C_dl_ is the double layer charging capacitance. The value of C_dl_ for electrodeposited Co/G NSs catalyst is 1.17 mF obtained from CV of different scan rates (Fig S3). Then, ECSA can be calculated from following relationship.

$${\rm{ECSA}}={{\rm{C}}}_{{\rm{dl}}}/{{\rm{C}}}_{{\rm{s}}}$$

Here C_s_ is the specific capacitance due to double layer of smooth surface of deposited sample on electrode. Values of C_s_ for different metal electrodes have been reported in literature for acidic and alkaline solutions lies in the range of 0.022−0.130 mF cm^−2^ for KOH and NaOH. The reported value of specific capacitance for cobalt surface is 0.043 mF cm^−2^ in 0.1 M KOH and consider it as a ‘typical’ value for such materials. The calculated electrochemically active surface area of Co/G NSs is 16 cm^2^ according to our calculations by using C_dl_ value.

## Electronic supplementary material

Supplementary Information
