# Supplementary material for: One-pot synthesis of graphene- cobalt hydroxide composite nanosheets (Co/G NSs) for electrocatalytic water oxidation
Source: Sci Rep. 2018 Sep 13;8:13772. doi: 10.1038/s41598-018-32177-9 (PMC6137037; doi:10.1038/s41598-018-32177-9)
Supplement: Supplementary file 1 — Supplementary Information [file 41598_2018_32177_MOESM1_ESM.docx]

**Supplementary Information**

**One-pot synthesis of graphene- cobalt hydroxide composite nanosheets (Co/G NSs) for electrocatalytic water oxidation**

Robab Mehmood^a,b^, Neelam Tariq^a^, Muhammad Zaheer^a*^, Fozia Bibi^b^, Zafar Iqbal^a^





**Figure S1**: Optimization of surfactant concentration (CTAB) for better yield of dispersed graphene by mechanical exfoliation method.


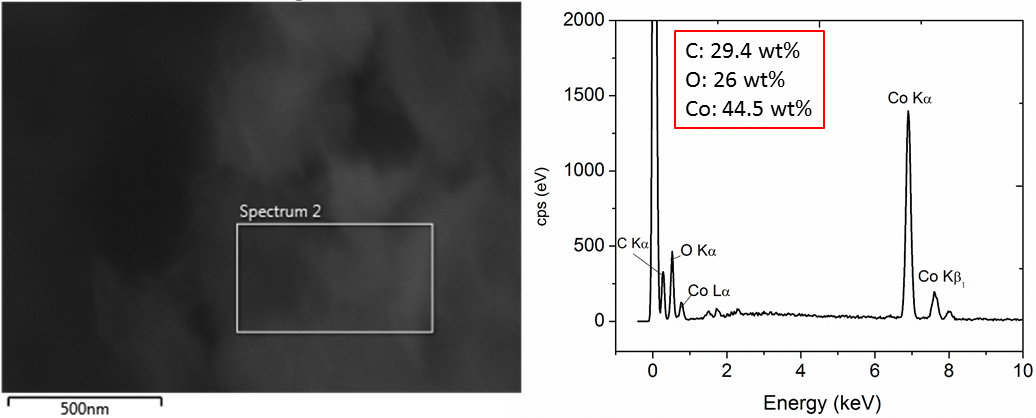


**Figure S2**. SEM image (left) and EDX of the selected region (right) for Co/G NSs confirms the presence of Co, O and C within the NSs.

**

**

**Figure S3**. Chronopotentiometric curve of Co/G NSs under applied current density of 10mA/cm^2^.





**Figure S4**: Cyclic Voltamogram (CV) of Co/G NSs at different scan rates for double layer capacitance measurement in 0.1 M KOH.





**Figure S5**: Voltamograms measured in non-Faradic region at 20 (black), 50 (red), 100 (green), 150 (blue) and 200 mV/sec (sky blue) scan rate while considering that all current is due to double layer capacitance


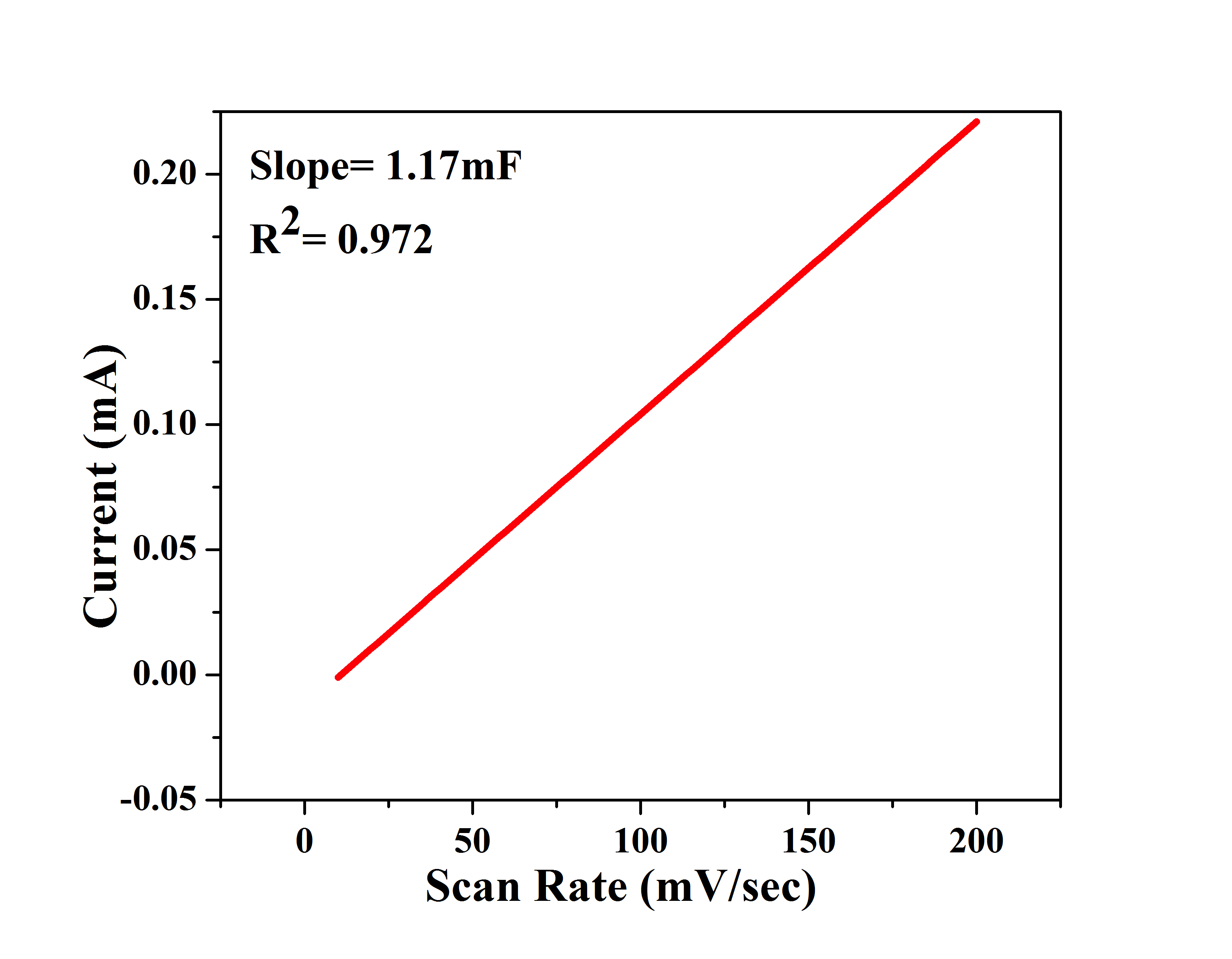


**Figure S6**: Plot of current (mA) vs Scan rate to find out double layer capacitance.

**Table S1**. Comparison of the activity of various cobalt-based catalysts for water oxidation

| **Serial no.** | **Catalyst** | **ɳ _@10mA/cm_^2^(mV)** | **Electrolyte (M)** | **Tafel slope (mV/dec)** | **References** |
| --- | --- | --- | --- | --- | --- |
| 01 | Ir_0.46_ Co_0.54_ O | 310 | 1M NaOH | 58.6 | S^3^ |
| 02 | Co_x_B | 292 | 1M KOH | 50.7 | S^4^ |
| 03 | SSUCo-900 | 337 | 1 MKOH | 428 | S^1^ |
| 04 | CoQDs/G | 370 | 0.1 MKOH | ~37 | S^5^ |
| 05 | Co/G NSs | 280 | 0.1 MKOH | 79.2 | **This work** |

**Table S2:** Comparison of electrochemical properties of different cobalt containing electrocatalysts

| **Serial no.** | **Catalyst** | **Mass activity (Ag^-1^)** | **TOFs(s^-1^)** | **ECSA(mA/cm^2^)** | **References** |
| --- | --- | --- | --- | --- | --- |
| 01 | SSUCo-900 | - | 0.034 | 18.9 | S^1^ |
| 02 | Co_3_O_4_@CoOSC | 234.0 | 0.0487 | - | S^2^ |
| 03 | Co/G NSs | 583.3 | 0.089 | 27 | **This work** |

**Table S3**. EIS data obtained from Nyquist plot of Co NPs@G

| **E/V** | **Electrodes** | **R_s_ (ohm)** | **R_p_ (ohm)** | **C_p_(ohm)** |
| --- | --- | --- | --- | --- |
| 1.51 | Co/G NSs | 72.83 ohm | 408.7ohm | 2.211 µF |

**References**

1. Zhang, G.; Wang, P.; Lu, W.-T.; Wang, C.-Y.; Li, Y.-K.; Ding, C.; Gu, J.; Zheng, X.-S.; Cao, F.-F., Co Nanoparticles/Co, N, S Tri-doped Graphene Templated from In-Situ-Formed Co, S Co-doped g-C3N4 as an Active Bifunctional Electrocatalyst for Overall Water Splitting. *ACS applied materials & interfaces* **2017,** *9* (34), 28566-28576.

2. Tung, C.-W.; Hsu, Y.-Y.; Shen, Y.-P.; Zheng, Y.; Chan, T.-S.; Sheu, H.-S.; Cheng, Y.-C.; Chen, H. M., Reversible adapting layer produces robust single-crystal electrocatalyst for oxygen evolution. *Nature communications* **2015,** *6*.

3. Yu, J.; Li, Q.; Chen, N.; Xu, C.-Y.; Zhen, L.; Wu, J.; Dravid, V. P., Carbon-coated nickel phosphide nanosheets as efficient dual-electrocatalyst for overall water splitting. **2016**.

4. Ma, X.; Wen, J.; Zhang, S.; Yuan, H.; Li, K.; Yan, F.; Zhang, X.; Chen, Y., Crystal Co x B (x= 1–3) Synthesized by a Ball-Milling Method as High-Performance Electrocatalysts for the Oxygen Evolution Reaction. *ACS Sustainable Chemistry & Engineering* **2017,** *5* (11), 10266-10274.

5. Govindhan, M.; Mao, B.; Chen, A., Novel cobalt quantum dot/graphene nanocomposites as highly efficient electrocatalysts for water splitting. *Nanoscale* **2016,** *8* (3), 1485-1492.
